# The Expression Pattern of microRNAs in Granulosa Cells of Subordinate and Dominant Follicles during the Early Luteal Phase of the Bovine Estrous Cycle

**DOI:** 10.1371/journal.pone.0106795

**Published:** 2014-09-05

**Authors:** Dessie Salilew-Wondim, Ijaz Ahmad, Samuel Gebremedhn, Sudeep Sahadevan, MD Munir Hossain, Franca Rings, Michael Hoelker, Ernst Tholen, Christiane Neuhoff, Christian Looft, Karl Schellander, Dawit Tesfaye

**Affiliations:** 1 Institute of Animal Science, Animal Breeding and Husbandry Group, University of Bonn, Bonn, Germany; 2 Department of Animal Breeding and Genetics, Bangladesh Agricultural University, Mymensingh, Bangladesh; China Agricultural University, China

## Abstract

This study aimed to investigate the miRNA expression patterns in granulosa cells of subordinate (SF) and dominant follicle (DF) during the early luteal phase of the bovine estrous cycle. For this, miRNA enriched total RNA isolated from granulosa cells of SF and DF obtained from heifers slaughtered at day 3 and day 7 of the estrous cycle was used for miRNAs deep sequencing. The results revealed that including 17 candidate novel miRNAs, several known miRNAs (n = 291–318) were detected in SF and DF at days 3 and 7 of the estrous cycle of which 244 miRNAs were common to all follicle groups. The let-7 families, bta-miR-10b, bta-miR-26a, bta-miR-99b and bta-miR-27b were among abundantly expressed miRNAs in both SF and DF at both days of the estrous cycle. Further analysis revealed that the expression patterns of 16 miRNAs including bta-miR-449a, bta-miR-449c and bta-miR-222 were differentially expressed between the granulosa cells of SF and DF at day 3 of the estrous cycle. However, at day 7 of the estrous cycle, 108 miRNAs including bta-miR-409a, bta-miR-383 and bta-miR-184 were differentially expressed between the two groups of granulosa cell revealing the presence of distinct miRNA expression profile changes between the two follicular stages at day 7 than day 3 of the estrous cycle. In addition, unlike the SF, marked temporal miRNA expression dynamics was observed in DF groups between day 3 and 7 of the estrous cycle. Target gene prediction and pathway analysis revealed that major signaling associated with follicular development including Wnt signaling, TGF-beta signaling, oocyte meiosis and GnRH signaling were affected by differentially expressed miRNAs. Thus, this study highlights the miRNA expression patterns of granulosa cells in subordinate and dominant follicles that could be associated with follicular recruitment, selection and dominance during the early luteal phase of the bovine estrous cycle.

## Introduction

Follicular development is the result of complex hormonal and biochemical synergies that could be activated or switched off within the follicular environment in a spatiotemporal manner. Follicles are the essential units of the ovary encompassing an oocyte along with one or several layers of granulosa cells [1]. The layer and number of granulosa cells may vary depending on the size and stage of follicular development. For instance, in primordial follicle, the non growing oocyte is enclosed by flattened single layer of pregranulosa cells [2], [3]. The primordial follicles then develop to the primary follicles by initiating growth and changing the single layer of granulosa cells to cuboidal morphology. The primary follicle in turn undergoes a continual differentiation and development of the granulosa cells and accompanied by enlargement of the oocyte volume which may gradually become Graafian follicle [3]. This programmed and complex transit of the primordial follicle into large sized antral follicle is mainly initiated by morphological transformation and functional differentiation of the granulosa cells. Therefore, when the follicles start to develop from state of resting pool, the oocytes continue to grow and the granulosa cells proliferate until the stage of preantral follicle [4]. Thus, during this critical periods, oocyte development is governed by paracrine interactions between the oocyte and the granulosa cells by which the oocyte modulates the growth and development of granulosa cells and vice-versa [2]. Therefore for symbiotic survival of both cell types, the granulosa and oocyte undergo a bidirectional communication by establishing a gap junction mediated syncytium [2], [3]. This bidirectional crosstalk between the oocyte and the somatic cell type (granulosa and theca) affects the hormonal production and the expression of genes associated with follicular development [5]. Hence, the growth, development and remarkable functional differentiation of the granulosa cells is one of the significant event that is required for follicle maturation [6]. The granulosa cells are indeed specialised in production of estradiol hormone, inhibin and activin [7]. Therefore, the fate of follicular growth and development is believed to be mainly determined by the growth and development of the granulosa cells [8].

In bovine follicular development is characterized by recruitment of a group of follicles in 2 or 3 follicular waves though high rate of growth is initiated only in one of the follicles which later on succeeds to be dominant over the others and becomes ovulatory [9]. However, the dominant follicle could not be ovulated when the follicular development is occurring during the luteal phase of the estrous cycle. Here the key question is to understand the mechanism how the follicular environment facilitates one follicle to become dominant and thus suppress the development of other follicles. This may lead to the hypothesis that the follicular microenvironment could have unique molecular signals which would affect differently the bidirectional crosstalk between the oocytes and the companion somatic cells in the subordinate and dominant follicles. In this regard, several signaling molecules including the TGF-beta superfamily members, follicle stimulating hormone receptor, luteinizing hormone receptor, cytochrome 450s *(CYP11A1, CYP17A1, CYP19A1)*, *GDF9, IGF-1, IGF-II, IGFBP2* and several of genes have been found to be altered in granulosa and/or theca cells depending on the size and stage of follicular development [10]–[14]. Abnormal expression of those developmentally related genes and gene products in the oocytes and supporting cells could then lead to cellular communication dysfunction and dysregulation of normal follicle recruitment and development [15]. However, the posttranscriptional regulatory mechanism of genes and gene products associated with follicular recruitment, selection and dominance during the luteal phase of the estrous cycle is still poorly understood.

MicroRNAs (miRNAs), which are 17–22 nucleotides small non-coding RNAs, are one of the molecular cues that are believe to be involved in posttranscriptional regulation of the spatial and temporal gene expression. The importance of miRNAs in the ovarian function, and follicular development has been described by several authors [15]–[18], but their presence, abundance and temporal expression in the subordinate and dominant follicles during the bovine luteal phase of the estrous cycle needs to be elucidated. Thus, here we employed a global analysis approach to uncover the miRNA enrichment and degradation in the granulosa cells of subordinate and dominant follicles during the early luteal phase of the estrous cycle using next generation high throughput miRNA sequencing technology.

## Materials and Methods

### Animal handling and management and estrous synchronization

A total of 13 Simmental healthy heifers were used for this study. All experimental heifers received similar total mixed ration and they were kept under the same farm and housing conditions. Handling and management of experimental animal was adhered to the rules and regulations of the German law of animal protection. Moreover, the experiment was approved by the Animal Welfare committee of the University of Bonn with proposition number 84–02.05.20.12.075. Prior to collection of experimental samples, all heifers used for the experiment were estrous synchronized as previously described [19]–[23]. Briefly, the heifers received intra muscular administration of 500 mg of the prostaglandin F_2α_ (PGF_2α_) analogue cloprostenol (Estrumate; Munich, Germany) twice within 11 days interval. Two days after each of the PGF_2α_ treatments, animals received 0.02 mg GnRH-analogue buserelin (Receptal) (Intervet, Boxmeer, the Netherlands). Common signs of estrus were monitored by visual observation and careful rectal palpation 3 days after the last PGF_2α_ injection. Following this, a total of 6 and 7 heifers were slaughtered at day 3 and 7 of the estrous cycle, respectively at nearby authorized abattoir. The ovaries were then collected from each experimental animal and transported to laboratory in a thermo-flask containing warm 0.9% NaCl saline solution.

### Follicle isolation and categorization

Immediately up on arrival in the laboratory within 1 hr after collection, the ovaries were washed once in 70% ethanol and three times in 0.9% NaCl solution. Each ovary was checked for the presence of cysts and corpus luteum. The ovaries from 3 cows (1 cow at day 3 and 2 cows at day 7 of the estrous cycle) were discarded from the experimental group. The ipsilateral or contralateral part of the ovary was noted based on the previous corpus luteum and the follicles were then isolated from each part of the ovary by blunt-dissection using scissors and forceps. The size of each follicle was measured using a caliper and then classified as subordinate and dominant follicles depending on their diameter as recommended earlier [24] with minor modifications. Briefly, at day 3 of the estrous cycle, follicles with follicular diameter of ≤6 mm (n = 43) were classified as subordinated follicle (SF) and follicles with a diameter of 8–10 mm (n = 9) were considered as dominant follicle (DF). On the other hand, at day 7 of the estrous cycle, follicles with a diameter of ≤8 mm (n = 58) were considered as SF and those with 9–13 mm diameter (n = 3) were categorized as DF.

### Granulosa and theca cell collection

Following classification of follicles as SF and DF, each of the follicles were cut at its top edge and the follicular contents were released into the plastic sterile culture dish. The follicular fluid remained in the plate after cumulus oocyte complex recovery was pooled according to the size of their corresponding follicles and centrifuged at 300 g for 10 min. The granulosa cells were sediment at the bottom of the falcon tube. The granulosa cell pellets were then washed twice with PBS without Ca^2^^+^ and Mg^2^^+^ and stored at −80°C for downstream analysis.

Theca cells were isolated after gently scraping the follicular wall with a blind edge of forceps and washed with Ca^2^^+^ and Mg^2^^+^ free PBS to remove any granulosa cell contamination. Then the theca cells (theca internal and theca externa) were pealed using forceps. After consecutive washings, the theca cells were transferred into 0.65 ml falcon tube containing RNA later and stored at −20°C for further analysis.

### RNA isolation from granulosa and theca cells

MicroRNA enriched total RNA was isolated from granulosa and theca samples using the miRNeasy Mini Kit (Qiagen GmbH, Hilden, Germany), following the manufacturers protocol. Any genomic DNA contamination was removed by performing on-column DNA digestion using RNase-free DNase (Qiagen GmbH, Hilden, Germany). The quantity and purity of RNA was determined with NanoDrop ND-800 (Thermo Fisher Scientific, Wilmington, DE, USA) whereas the RNA integrity was evaluated using the RNA 6000 Nano Kit and 2100 Bioanalyzer (Agilent, Palo Alto, CA, USA). The granulosa RNA samples with the absorbance wavelength ratio (A260/A280) of ≥1.9 and the RNA integrity number of ≥7 were used for miRNAs deep sequencing. A total of 12 granulosa samples (three biological replicates of granulosa cells from SF or DF at day 3 or day 7 of the estrous cycle) were used for miRNA deep sequencing. However, the total RNA samples from theca cells were used for measuring the expression of candidate miRNAs generated from granulosa cells.

### Illumina miRNA library preparation and sequencing from granulosa cells

MiRNA library preparation and sequencing of the samples were performed by GATC BioTech AG (Konstanc, Germany) using the Illumina® TruSeq™ Small RNA sample preparation protocol, which takes the advantage of the natural structure common to most known microRNA molecules with minor modification. Briefly; to enrich miRNA population, the specific 3′RNA adapters were ligated to the 1 µg total RNA followed by ligation of RNA 5 Adapter (RA5). Following this, reverse transcription was performed using RNA RT primers (RTP). The sequences of the adapters and RTP are described in table S1. PCR amplification and adding unique index sequences to cDNA were performed using RNA PCR Primers (RP1) (Table S2). The amplified cDNAs were gel purified and approximately 145–160 bands that correspond to the adapter-ligated constructs derived from the 19–22 nucleotides were recovered using clean scalpel. The miRNA enriched sequencing libraries were pooled together and sequencing was performed on a HiSeq 2000 in single read mode with a read length of 50 bases. Base calling, data filtering and index sorting was performed by the CASAVA Pipeline version 1.8.0. The raw sequence data was generated as fastq.

### Quality assessment of raw sequencing data and adapter trimming

Quality evaluation of the raw sequence data was performed using FastQC, a free available sequence analysis tool, (http://www.bioinformatics.babraham.ac.uk/publications.html). For this, the raw fastq data was imported into the program and the basic statistics, the sequence quality, quality score, sequence content and GC content were evaluated perbase basis. The raw sequence data that satisfies the basic requirements of quality parameters were used for downstream analysis. The adapters, PCR primers, non informative sequences, sequence reads with phred score lower than 18 and sequences shorter than 18 bp were removed from downstream analysis using the cutadapt software (http://code.google.com/p/cutadapt/). The raw sequencing reads and the processed data have been deposited in NCBI's Gene Expression Omnibus with GEO accession number GSE55987.

### Sequence alignment and identification of known miRNAs

Sequence alignment and detection of known and novel microRNAs were performed using miRDeep2 software algorithm [25]. Prior to performing sequence alignment, the bovine reference genome release 72 (UMD 3.1) was retrieved from Ensembl Genome Browser (ftp://ftp.ensembl.org/pub/release-72/fasta/bos_taurus/dna/) and indexed using Bowtie 2–2.1.0, a freely available tool for aligning sequencing reads to long reference sequences (http://bowtie-bio.sourceforge.net/bowtie2/index.shtml). The sequence reads were then aligned to the indexed bovine reference genome. Those sequence reads aligned to the bovine reference genome were then blasted against the bovine matured and precursor miRNAs and matured miRNAs of other species (human, rat and mouse) obtained from miRBase v.20, (http://www.mirbase.org/).

### Novel miRNA predictions

Novel miRNAs were predicted from mature, star and loop sequence according to the RNAfold algorithm using miRDeep2 [25], [26]. RNAfold was used for prediction of RNA secondary structures. In addition, we also calculated the estimated probability that the sequence to be true a positive novel miRNA candidate. Apart from this, the sequence should not be mapped to the rRNA or tRNA. A sequence was considered as a novel miRNA when it was not matched to any of the known miRNA in the mirBase.

### Analysing differentially expressed miRNAs

Following detection and identification of miRNAs, the expression level of those miRNAs was compared between the granulosa samples of SF and DF to understand whether accumulation or degradation of miRNA is associated with follicular selection, recruitment and dominance. For this, four comparisons were performed. (1) To understand whether accumulation or degradation of miRNAs is associated with follicular selection and recruitment in early luteal phase, the expression pattern of miRNAs were compared between the granulosa cells of SF and DF at day 3 of the estrous cycle. (2) To investigate whether the miRNA expression is associated with follicular selection and dominance during the early luteal phase, the expression pattern of miRNAs was compared between granulosa cells of SF and DF at day 7 of the estrous cycle. (3) To understand the temporal miRNAs expression dynamics in DF during the luteal phase of the bovine estrous cycle, the miRNA expression pattern of granulosa cells in DF samples collected at day 7 was compared to the DF samples collected at day 3 of the estrous cycle. (4) The miRNA expression pattern of the granulosa cells of SF was also compared between day 3 and day 7 of the estrous cycle to identify the miRNAs that could be changing in SF follicles during the early luteal phase of the estrous cycle. Differentially expressed miRNAs were analysed from read counts of the samples using R software (http://cran.r-project.org) and DESeq2 package [27]. For this, normalization of the data was performed in such a way that the raw read count of each miRNA was multiplied by the sample size factor which was calculated as the median of ratios of observed count. For each miRNA in each granulosa sample, the observed count is the ratio of raw count for each miRNA to the geometric mean across the samples. The Benjamini–Hochberg procedure of false discovery rate adjustment [28] and a negative binomial distribution based method integrated in DESeq2 were employed to identify differentially expressed miRNAs between granulosa cells. At the end of analysis, miRNAs with log_2_ fold change difference ≥1.0, with *p* value ≤0.05 and false discovery rate (FDR) ≤10% were considered as differentially expressed miRNAs. The heatmaps and clustering analysis of the differentially expressed miRNAs were generated using PermutMatrix [29].

### Target gene prediction of differentially expressed miRNAs and their functional annotation

The functional annotation of differentially expressed miRNAs was analysed based on the functional annotation of their potential target genes. The predicted target genes were identified using miRecords http://mirecords.biolead.org/, an online animal miRNA-target interaction tool which integrates 12 miRNA-target prediction tools, including DIANA-microT, MicroInspector, miRanda, MirTarget2, miTarget, PicTar, PITA, and TargetScan. The target genes which were predicted by at least four prediction tools were selected and submitted to DAVID Functional Annotation Bioinformatics Microarray Analysis tool [30] and the potential KEGG, Panther and Reactome pathways significantly enriched by target genes of each miRNAs were identified.

### Validation of differentially expressed miRNAs using quantitative real time polymerase chain reaction (qPCR)

The expression levels of 12 differentially expressed miRNAs were further validated using qPCR. For that, 88 ng miRNA enriched total RNA was reverse transcribed using universal cDNA synthesis kit (Exiqon) following the manufactures protocol. Briefly, a 20 µl reaction mix consisting of total RNA, reaction buffer, enzyme mixes and nuclease-free water was incubated at 42°C for 60 min, followed by 5 min incubation at 95°C. At the end of reaction period, the resulting cDNA was diluted 40× in RNAse free water. The qPCR was then performed in 20 µl reaction volume containing cDNA of each sample mixed with reverse and forward primer of each miRNA, PCR master mix (SYBR Green, thermo stable DNA polymerase) and nuclease-free water in the StepOnePlus™ Real-Time PCR Systems (Applied Biosystems, Foster City, CA). The real time PCR thermocycler parameter was set to 95°C of denaturation for 10 min followed by 40 cycles at 95°C for 15 s and 60°C for 30 s. At the end of 40 cycles, the melting curve was analysed at 95°C for 15 s, 60°C for 1 min and the temperature was increased at the rate of 0.3/s until it reached to 95°C and the reaction was incubate at 95°C for 15 s. At the end of the reaction, the qPCR data with acceptable dissociation curve and the amplification plot was used for further quantification analysis. The data was analysed using the delta threshold cycle (delta Ct) method. The delta Ct values were generated after the expression of each miRNA was normalized against the arithmetic mean expression value of U6 and 5S. The resulting delta Ct values were used to generate the heatmap using PermutMatrix [29]. The within similarities of biological replicates and differences between samples of the qPCR data was then compared with the data generated by next generation sequencing.

### Characterization of the expression of candidate miRNAs in theca cells

Following identification of differentially expressed miRNAs between the granulosa cells of SF and DF, we opted to understand whether the expression observed in granulosa cell could follow a similar pattern in theca cells. For this, 8 differentially expressed miRNAs both at day 3 and day 7 of the estrous cycle, namely bta-miR-708, bta-miR-214, bta-miR-335, bta-miR-221, bta-miR-21-3p, bta-miR-21-5p, bta-miR-155 and bta-miR-34c and 3 miRNAs; bta-miR-195, bta-miR-365-5p and bta-miR-409 which were differentially expressed only at day 7 of the estrous cycle and 1 miRNA; bta-miR-34c that was differentially expressed at day 3 of the estrous cycle were analysed in theca cells. For this, the miRNA enriched total RNA was isolated from theca cells of SF and DF whose granulosa cells were used for the next generation sequencing was subjected to cDNA synthesis and qPCR analysis. The qPCR reaction, the thermo cycler parameters and the data analysis was performed exactly as indicated above. The level of activation or repression of miRNA expression in theca cells of SF relative to DF was determined using the formula 2^-**ΔC**T^ [31].

## Results

### Identification of known miRNAs in granulosa cells of subordinate and dominant follicles at day 3 and day 7 of the estrous cycle

To understand the miRNAs enrichment and degradation during the luteal phase of the bovine estrous cycle, twelve (three biological replicates for each sample) miRNA libraries were prepared from granulosa cells of subordinate (SF) and dominant (DF) at day 3 and day 7 of the estrous cycle using Illumina HiSeq 2000 and sequence reads with maximum 50 base-pair (bp) length were obtained. At day 3 of the estrous cycle, the raw sequence data within the biological replicates of SF groups ranges from 3.9 to 7.7 million reads, whereas in DF, the read count ranges from 8.7 to 9.5 million reads. Similarly, at day 7 of the estrous cycle, raw sequence data in granulosa cells of SF was between 6.7 and 8.1 million reads whereas in DF groups the raw read counts was between 7.5 and 9.2 million. However, after filtering adapters, PCR primers, non informative sequences and sequences shorter than 18 bp, the mean read counts of SF and DF samples at day 3 of the estrous cycle were 1.5±0.32 and 1.9±0.18 million reads, respectively. Similarly, at day 7 of the estrous cycle, the mean read count was 1.9±0.18 and 1.6±0.15 in granulosa samples of SF and DF, respectively.

**Figure 1:**
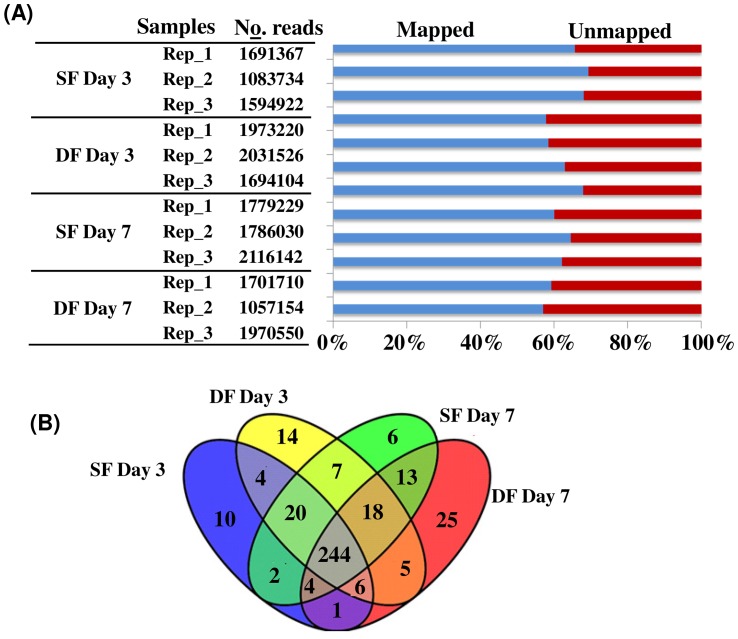
Detected miRNAs in granulosa cells of DF and SF at day 7 and day of the oestrous cycle. (A) The number of filtered sequence reads obtained in each biological replicates of SF and DF granulosa samples at day 3 and day 7 of the estrous cycle and the percentage of aligned sequences to the bovine reference genome (A). The blue and the red colour indicate the percentage of mapped and unmapped sequence reads, respectively. Rep_1, Rep_2, and Rep_3 indicate the biological replicates in each sample group. (B) Venn diagram showing the number of known miRNAs detected uniquely or commonly in SF and DF granulosa samples at day 3 and day 7 of the estrous cycle. SF Day 3 and DF Day 3 indicate the subordinate and dominant follicles, respectively at day 3, while SF Day 7 and DF Day 7 indicate the subordinate and dominant follicles, respectively at day 7 of the estrous cycle. The Venn diagram was drawn using Venny software, a freely available online tool http://bioinfogp.cnb.csic.es/tools/venny/.

**Figure 2:**
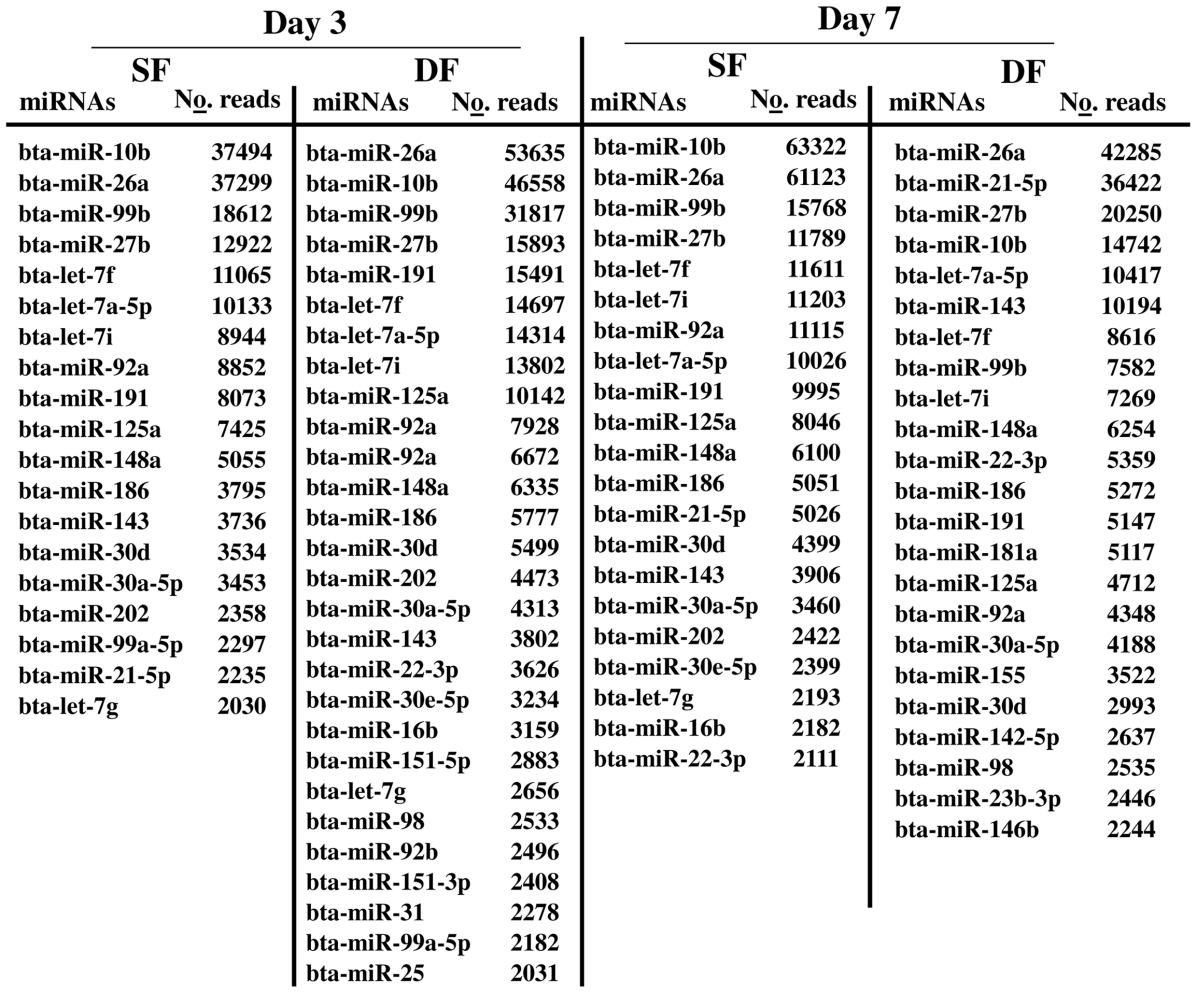
The top most abundant miRNAs with >2000 read counts in granulosa samples of SF or DF at day 3 and/or day 7 of the estrous cycle. No. reads describe the average number of read counts aligned to each miRNA.

Sequence alignment of filtered reads indicated that 59.5% and 64.2% of the sequence reads in SF and DF, respectively at day 3 of the estrous cycle and 59.8% and 67.7% of the total filtered reads in SF and DF, respectively at day 7 of the estrous cycle were aligned to the bovine reference genome (Figure 1A). Following this, we used the miRDeep2.pl of mirDeep2 script to identify known and novel miRNAs. Accordingly, miRNAs with at least 1 raw read count in at least 2 of the three biological replicates were considered as detected miRNAs. Thus, 43025 and 4400226 reads of SF and DF, respectively at day 3 of the estrous cycle and 411736 and 358128 sequence reads of SF and DF, respectively at day 7 of the estrous cycle were matched to the known bovine miRNAs available in miRbase. Based on this analysis, a total of 291 and 318 known miRNAs were detected in granulosa cells of SF and DF, respectively at day 3 of the estrous cycle and 274 miRNAs were commonly expressed in both granulosa cell groups. On the other hand, 314 and 316 known miRNAs were detected in granulosa cells of SF and DF, respectively at day 7 of the estrous cycle of which 279 were detected in both granulosa cell groups. In addition, taking all data together, a total of 244 miRNAs were commonly detected in all granulosa cells in both follicles sizes at both days of the estrous cycle (Figure 1B), of which 15 miRNAs were dominantly abundantly by ≥2000 reads (Figure 2). Among these, bta-miR-10b, bta-miR-26a, bta-miR-99b, bta-miR-27b, let-7 families (bta-let-7f, and bta-let-7a-5p) and bta-miR-92a appeared ≥10000 read counts in each sample (Figure 2).

### Identification of differentially expressed miRNAs between granulosa cells of subordinate and dominant follicles at day 3 of the estrous cycle

**Figure 3:**
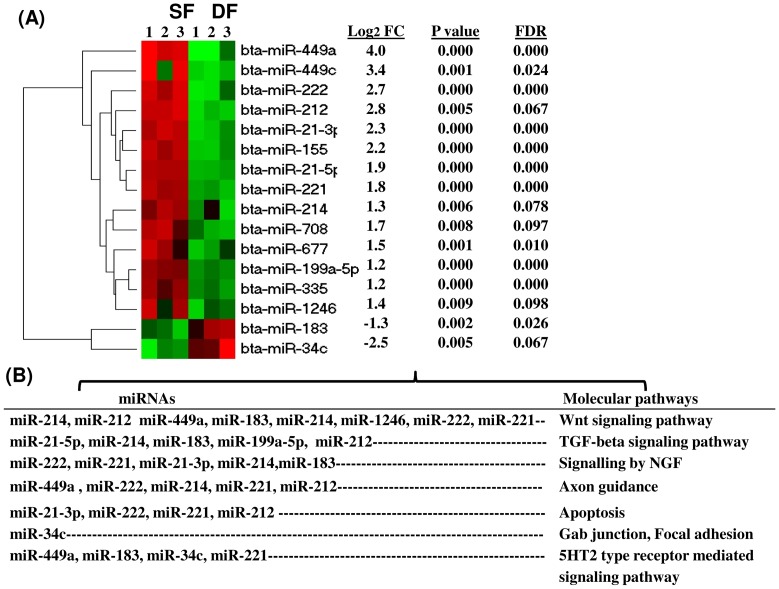
Differentially expressed known miRNAs between the granulosa cells of SF and DF at day 3 of the estrous cycle. (A) The hierarchical clustering of differentially expressed miRNAs along with their average expression difference (FC = log_2_ fold change), *p* value, and false discovery rate (FDR). Positive and negative FC values indicate up and downregulation of miRNA, respectively in SF compared to DF granulosa cells. The red and green colours designate high and low expression of miRNAs, respectively. B) The pathways enriched by genes targeted by differentially expressed miRNAs.

To understand whether miRNAs expression is altered during follicular recruitment and selection, during the early luteal phase, we investigated the expression pattern of miRNAs at day 3 of the estrous. Therefore, following detection of the known miRNAs, their expression difference between the granulosa cells derived from SF and DF was analysed using the DESeq2 by comparing the log_2_ transformed read counts of granulosa cells of SF and DF. The expression pattern of 280 miRNAs was found to be altered by ≥1.5 fold changes compared to the granulosa cells of the DF of which 57% were activated while the rest 43% were repressed in the SF groups. However, when the selection parameters were limited to log_2_ fold change ≥1.0 (absolute fold change ≥2), *p* value <0.05 and FDR <10%, only 16 known miRNAs were found to be significantly differentially expressed between the granulosa cells of SF and DF groups. Among these, the expression level of 14 miRNAs including bta-miR-449a, bta-miR-449c, bta-miR-212, bta-miR-222, bta-miR-21-3p and bta-miR-155 were increased while the expression level of 2 miRNAs (bta-miR-183, bta-miR-34c) was decreased in SF groups. The hierarchical clustering, the average expression, the *p* value and the FDR values of the differentially expressed miRNAs are described in figure 3A. To uncover, the role of those differentially expressed miRNAs, their target genes were predicted using miRecords tool and significantly enriched pathway (P≤0.05) by those genes was analysed using DAVID Bioinformatics Resources 6.7 (http://david.abcc.ncifcrf.gov/). Accordingly, the Wnt signaling pathway, TGF-beta signaling pathway, signaling by nerve growth factor (NGF), axon guidance, apoptosis and 5HT2 type receptor mediated signaling pathway were identified as a potential pathways enriched by target genes of those miRNAs (Figure 3B).

### Differentially expressed miRNAs between granulosa cells of subordinate and dominant follicles at day 7 of the estrous cycle

**Figure 4:**
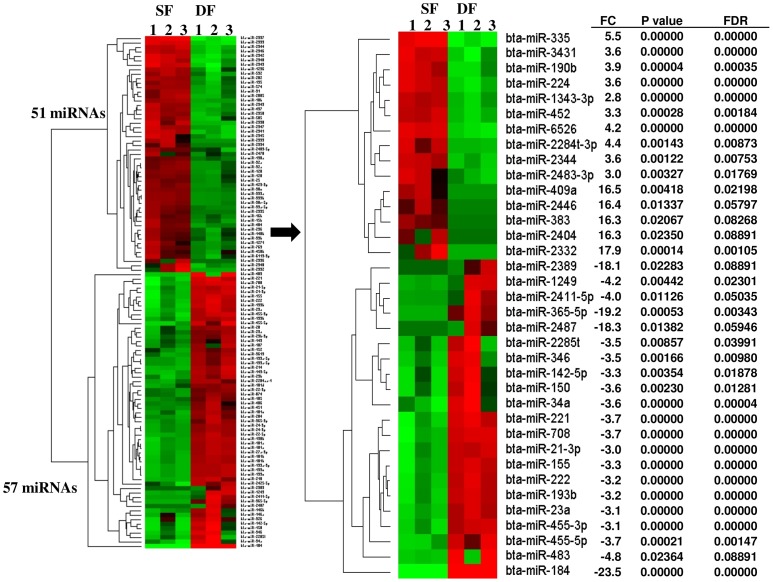
Differentially expressed miRNAs between granulosa cells of SF and DF at day 7 of the estrous cycle. (A) The expression patterns and hierarchical clustering of 108 differentially expressed miRNAs between granulosa cells of SF and DF. The numbers 1, 2, 3 under SF and DF indicate the biological replicates. (B) The expression patterns and hierarchical clustering of top 36 differentially expressed miRNAs along with their average expression difference (FC = log_2_ fold change), p values and false discovery rate (FDR). The red and green colours indicate high and low expression, respectively. Positive and negative FC values indicate up and downregulation of miRNA, respectively in SF compared to the DF granulosa cells.

To uncover whether the degradation or enrichment of miRNAs is associated with follicular selection and dominance during the early luteal phase, the expression profiles of miRNAs were then compared between granulosa cells of SF and DF at day 7 of the estrous cycle using the DESeq2 package. The results revealed that a total of 272 known bovine miRNAs exhibited ≥1.5 fold change differences between the two granulosa cell groups of which 49% of those miRNAs were enriched while the rest 51% of them were reduced in the granulosa cells of SF. However, when the miRNAs were filtered based on the criteria of log_2_ fold change ≥1.0, *p* value ≤0.05, FDR ≤10%, a total of 108 known miRNAs were differentially expressed between the granulosa cells of SF and DF, of which the expression level of 51 miRNAs were increased while the expression level of 57 miRNAs were reduced in the granulosa cell of SF. Among these, bta-miR-2332, bta-miR-409a, bta-miR-2446, bta-miR-383, bta-miR-2404 and bta-miR-335 were not detected in granulosa cells of DF. On the other hand, from 57 downregulated miRNAs, bta-miR-184, bta-miR-365-5p, bta-miR-2487 and bta-miR-2389 were not absent in granulosa cells of SF. In addition, the expression of 12 miRNA families including miR-130 (*a, b*), bta-miR-181 (*a, b, c, d*), bta-miR-199 (*a-3p, a-5p, b, c*), bta-miR-2285 (*k, t*), bta-miR-2411 (-*3p, -5p*), bta-miR-2483 (-*3p, -5p*), bta-miR-29 (*a, b*), bta-miR-339 (*a, b*), bta-miR-365 (-*3p, -5p*), bta-miR-455 (-*3p, -5p*), bta-miR-92, and bta-miR-99 (*a, -5p, b*) were differentially expressed between the granulosa cells of SF and DF (Table 1). The expression and hierarchical clustering of all differentially expressed miRNAs and the top 36 ones are described in figure 4A and 4B, respectively. Moreover, the list of 108 differentially expressed miRNAs with their corresponding fold change, *p* values and FDR is provided in table S3.

**Table 1:** MiRNA families co-expressed or co-repressed in granulosa cells of SF compared to DF at day 7 of estrous cycle.

miRNA	FC	P value
bta-miR-130a	1.0	0.0001
bta-miR-130b	−2.5	<0.0001
bta-miR-181a	−2.4	<0.0001
bta-miR-181b	−2.3	<0.0001
bta-miR-181c	−1.8	<0.0001
bta-miR-181d	−1.5	0.074
bta-miR-199a-3p	−2.5	<0.0001
bta-miR-199a-5p	−2.1	<0.0001
bta-miR-199b	−2.4	<0.0001
bta-miR-199c	−2.9	<0.0001
bta-miR-2285k	2.6	<0.0001
bta-miR-2285t	−3.5	0.0085
bta-miR-2411-3p	2.4	0.0055
bta-miR-2411-5p	−4.0	0.0112
bta-miR-2483-3p	3.0	0.0032
bta-miR-2483-5p	1.2	0.0212
bta-miR-29a	−1.4	0.0001
bta-miR-29b	−2.2	0.0018
bta-miR-339a	1.6	<0.0001
bta-miR-339b	1.6	<0.0001
bta-miR-365-3p	−1.9	<0.0001
bta-miR-365-5p	−19.2	0.0005
bta-miR-455-3p	−3.1	<0.0001
bta-miR-455-5p	−3.8	0.0002
bta-miR-92a	1.4	<0.0001
bta-miR-92b	2.2	<0.0001
bta-miR-99a-5p	1.3	<0.0001
bta-miR-99b	1.1	0.002

*FC =  fold change in log_2_ scale. Positive FC values indicate upregulation and negative FC values indicate downregulation of miRNAs in SF compared to DF.*

**Figure 5:**
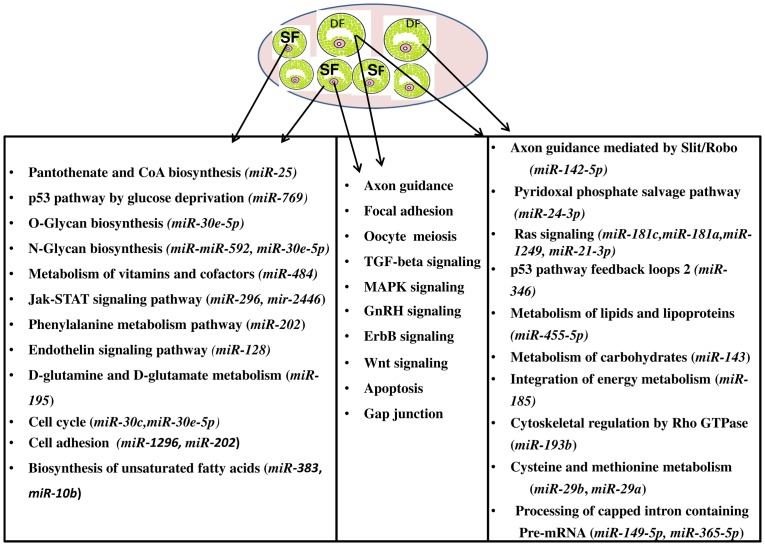
Significant molecular pathways (P≤0.05) enriched by genes targeted by differentially expressed miRNAs between the granulosa cells of SF and DF at day 7 of the estrous cycle. Pathways enriched by genes potentially targeted only by miRNAs increased in SF are indicated in the left box, pathways enriched by genes potentially targeted by both miRNAs repressed and activated in SF are shown in the middle box while pathways enriched by genes potentially targeted only by miRNAs upregulated in DF but repressed in SF are described in the right box.

**Figure 6:**
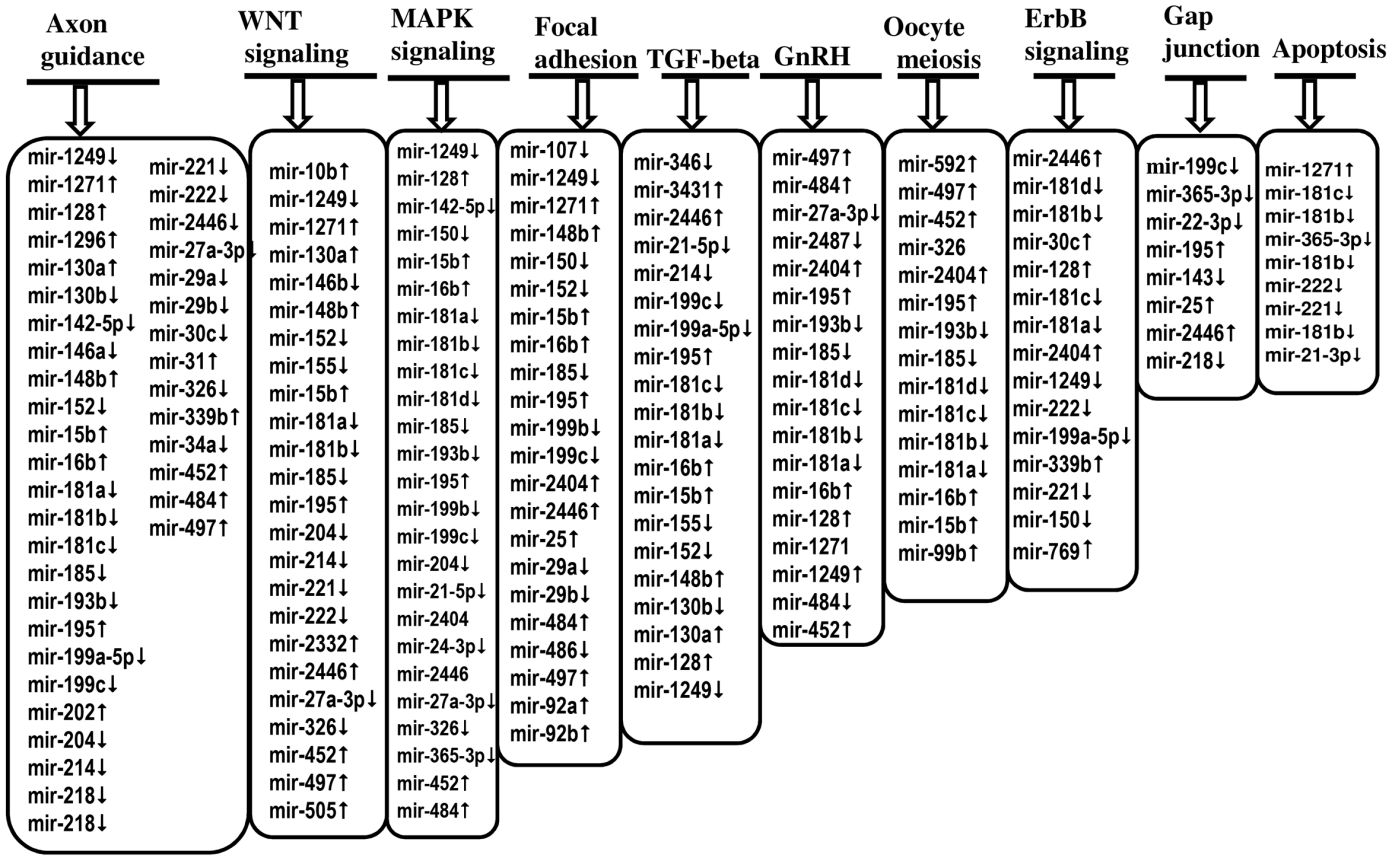
The list of differentially expressed miRNAs whose target genes are enriched (p≤0.05) in Wnt signaling, GnRH signaling, MAPK, signaling, oocyte meiosis, TGF-beta signaling, focal, adhesion, ErbB, gap junction, axon guidance and apoptosis. (↑) indicates increased expression while (↓) depicts the reduction of miRNA expression in granulosa cells of SF compared to DF groups at day 7 of the estrous cycle.

After detecting the differentially expressed miRNAs, the signaling pathways and functions that could be regulated by genes potentially targeted by differentially expressed miRNAs were also investigated using a bioinformatics analysis. The predicted target genes of each miRNAs were uploaded to the DAVID Bioinformatics Resources version 6.7 tool and significantly (p≤0.05) enriched molecular pathways were identified. The results showed that among 108 miRNAs, the targets of 84 miRNAs were found to be involved in metabolic and signaling molecular pathways. Metabolic pathways (panthothenate and CoA biosynthesis, O-glycan and N-glycan biosynthesis, metabolism of vitamins and cofactors, D-glutamine and glutamate metabolism, biosynthesis of unsaturated fatty acids), Jak-STAT and cell cycle pathways were enriched by genes potentially targeted by miRNAs enriched in granulosa cells of SF, whereas the Ras signaling pathway, lipids, lipoproteins, cysteine, methionine and carbohydrates metabolic pathways were enriched by genes potentially targeted by miRNAs repressed in granulosa cells of SF. Interestingly, axon guidance, Wnt signaling pathway, GnRH signaling pathway, MAPK-signaling, oocyte meiosis, TGF-beta signaling pathway and focal adhesion were enriched by genes targeted by miRNAs either enriched or repressed in granulosa cells of SF (Figure 5, Figure 6).

### Commonly differentially expressed miRNAs between the granulosa cells of subordinate and dominant follicle both at day 3 and day 7 of the estrous cycle

**Figure 7:**
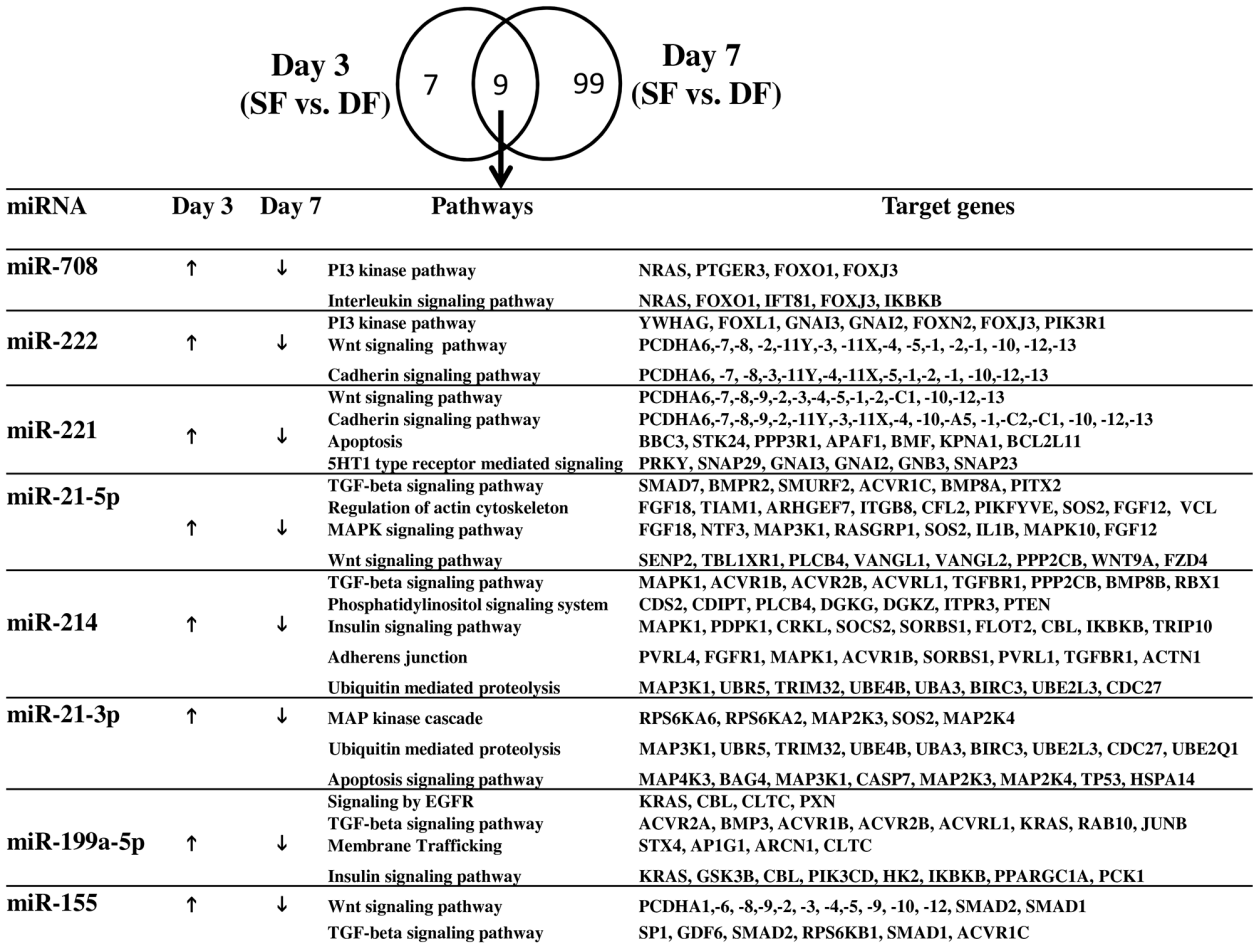
Uniquely and commonly differentially expressed miRNAs between the granulosa cells of SF and DF at day 3 and day 7 of the estrous cycle and their pathways enriched by their potential target genes. (↑) shows upregulation while (↓) indicate downregulation of commonly differentially expressed miRNAs in the granulosa cells of SF compared to DF at day 3 or day 7 of the estrous cycle.

Compared to day 3, the number of differentially expressed miRNAs between the granulosa cells of SF and DF was higher at day 7 of the estrous cycle (16 vs. 108 miRNAs). However, 9 miRNAs were commonly differentially expressed between granulosa cells of SF and DF both at day 3 and 7 of the estrous cycle. Interestingly, 8 miRNAs namely (bta-miR-199a-5p, bta-miR-214, bta-miR-708, bta-miR-221, bta-miR-21-5p, bta-miR-155, bta-miR-21-3p and bta-miR-222) were found to be enriched in SF at day 3 of the estrous cycle but at day of 7 the estrous cycle those miRNAs were repressed in SF compared to the DF groups (Figure 7). Moreover, *Insilco* analysis of the function of the miRNAs via their target genes indicated that these miRNAs were found to be involved in 2 or more pathways. For instance, miR-221 and mir-222 were found to be involved in Wnt and cadherin signaling pathways by targeting protocadherin alpha family of genes (*PCDHA1,-2,-3,-4, -5,-6,-7,-8* etc.). In addition, miR-221, is also involved in apoptosis and 5HT1 type receptor mediated signaling by targeting genes involved in these pathways. Similarly, mir-21-5p was found to be involved in TGF-beta signaling pathway, regulation of actin cytoskeleton and MAPK signaling pathway and mir-199a-5p was found to be involved in TGF-beta signaling pathway, epidermal growth factor receptor (EGFR) signaling, membrane trafficking and insulin signaling pathway (Figure 7).

### Temporal enrichment or degradation of miRNAs in granulosa cells of DF during the early luteal phase of the estrous cycle

**Figure 8:**
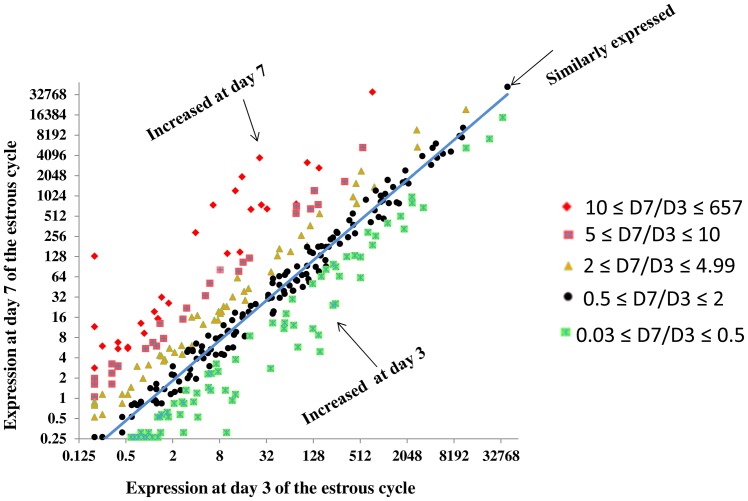
Scatter plot showing the read count ratio of 357 miRNAs between day 7 and day 3 in the granulosa cells of DF.

**Figure 9:**
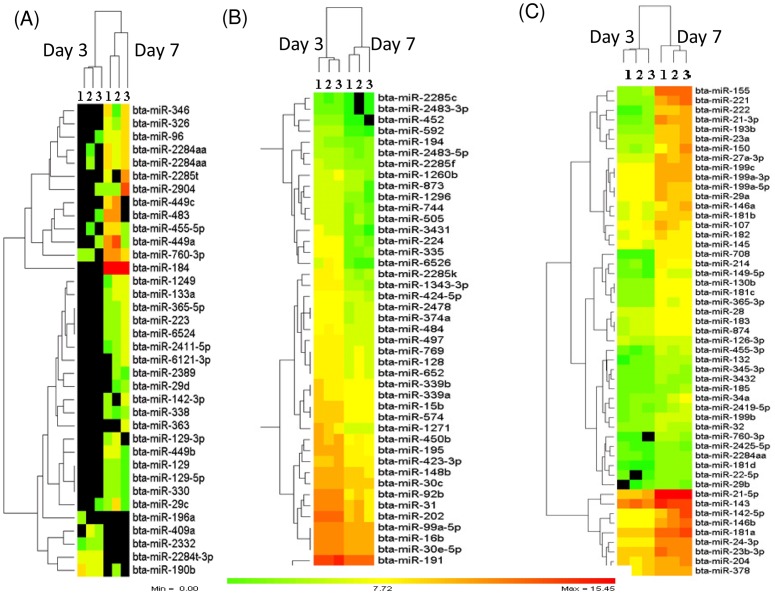
The heatmaps and the hierarchical clustering depicting the expression patterns of differentially expressed miRNAs in granulosa cells of DF between day 3 and 7 of the estrous cycle. (A) The expression patterns of miRNAs detected only at day 3 (top) or at day 7 (bottom) of the estrous cycle in granulosa cells of DF. (B) The expression patterns of miRNAs detected both at day 3 and at day 7 of the estrous cycle but significantly increased in the former group. (C) The expression patterns of miRNAs expressed both at day 3 and at day 7 of the estrous cycle but significantly increased in the later group. The colour scale shows the log_2_ transformed expression values. Zero colour scale indicates miRNAs with ≤1 average read count. Numbers, 1, 2 and 3 on the heatmaps describe the number of biological replicates used in each sample group. Day 3 and Day 7 indicate the stages of the estrous cycle post estrus.

**Figure 10:**
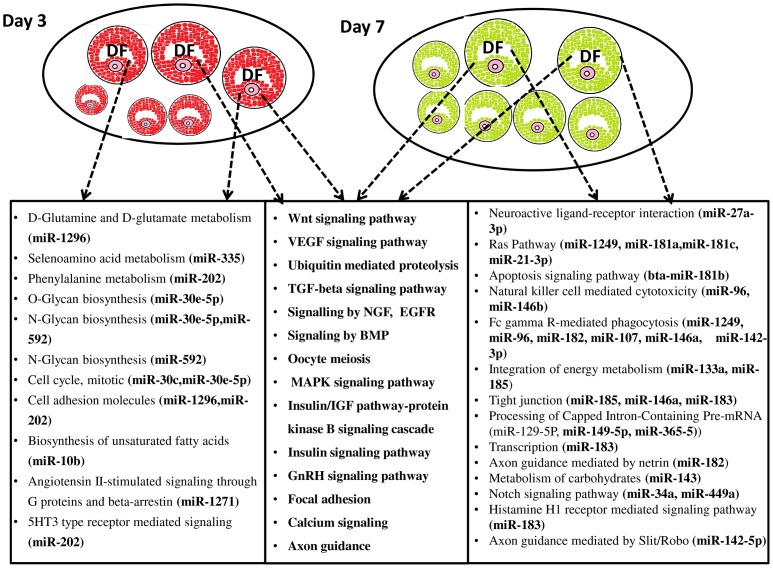
Graphical illustration of DF follicles and molecular pathways enriched by genes targeted by differentially expressed miRNAs between day 3 and day 7 of the estrous in granulosa cells of DF. Pathways significantly (P≤0.05) enriched by genes potentially targeted only by miRNAs enriched at day 3 are indicated in the left box. Pathways enriched by genes potentially targeted by miRNAs increased at day 3 and day 7 of the estrous cycle are listed in the middle box and pathways enriched by genes potentially targeted only by miRNAs increased at day 7 of the estrous cycle are described in the right box. DF, dominant follicle, Day 3 and Day 7 indicate the stages of the estrous cycle post estrus.

We determined the temporal miRNA expression patterns in granulosa cells of DF between day 3 and day 7 of the estrous cycle to uncover the miRNAs expression dynamics in dominant follicles during the luteal phase of the bovine estrous cycle. Thus, after normalization of the read counts, the number of detected miRNAs were quite similar in both granulosa cells of DF at day 3 (n = 318) and day 7 of the estrous cycle (n = 316) of which 262 miRNAs were commonly detected at both time points. The scatter plot of all those miRNAs is indicated in figure 8 to depict the overall expression difference. In addition to this, we also analysed the miRNAs whose expression is significantly different in DF between the two time points. Accordingly, a total of 131 miRNAs were differentially expressed between day 3 and day 7 of the estrous cycle of which 51 and 80 miRNAs were increased at day 3 and at day 7 of the estrous cycle, respectively (Figure 9). Among miRNAs increased at day 7 of the estrous cycle, bta-miR-2389, bta-miR-29d, bta-miR-363, bta-miR-1249, bta-miR-338, bta-miR-129-3p, bta-miR-129-5p, bta-miR-129, bta-miR-142-3p, bta-miR-449b, bta-miR-2285t and bta-miR-346 were not detected at day 3 (Figure 9A). On the other hand, among the miRNAs that were repressed at day 7 of the estrous cycle bta-miR-409a, bta-miR-2332 and bta-miR-196a were detected at day 3 but disappeared at day 7 of the estrous cycle (Figure 9A). Others namely, bta-miR-143, bta-miR-99b, bta-miR-191, bta-miR-16b, bta-miR-99a-5p and bta-miR-30e-5p were expressed by >500 reads counts both at day 3 and day 7 of the estrous cycle. However, the expression pattern of these miRNAs was significantly decreased at day 7 compared to day 3 of the estrous cycle. The expression pattern of miRNAs expressed both at day 3 and day 7 but downregulated at later groups is shown in figure 9B and those upregulated are indicated in figure 9C. Moreover, the expression of 13 miRNA families including bta-miR-29 (a, b, c, d), bta-miR-449 (a, b, c), bta-miR-181 (a, b, c, d), bta-miR-455 (-3p, -5p), bta-miR-99 (b, a-5p) and bta-miR-2483 (-5p, -3p) were co-overexpressed or co-repressed at day 7 compared to day 3 (Table 2). In addition, target gene prediction and pathway analysis showed that including transcription, the neuroactive ligand-receptor interaction, apoptosis signaling pathway, natural killer cell mediated cytotoxicity, Fc gamma R-mediated phagocytosis, tight junction, metabolism of carbohydrates, notch signaling pathway, axon guidance mediated by Slit/Robo and axon guidance mediated by netrin were enriched by genes targeted by miRNAs elevated at day 7, whereas the vitamin D and Phenylalanine metabolism, O-glycan and N-glycan biosynthesis, mRNA metabolic process, cell cycle and biosynthesis of unsaturated fatty acids were enriched by genes targeted by miRNAs downregulated at day 7 the estrous cycle. On the other hand, Wnt signaling, vascular endothelial growth factor (VEGF) signaling, TGF-beta signaling, epidermal growth factor receptor (EGFR) signaling, oocyte meiosis, MAPK signaling, GnRH signaling, focal adhesion, axon guidance, angiogenesis, calcium signaling and ubiquitin mediated proteolysis were enriched by genes potentially targeted by both down and upregulated miRNAs (Figure 10, Table S4).

**Table 2:** MiRNA families co-expressed or co-repressed in granulosa cells of DF at day 7 compared to day 3 of the estrous cycle.

miRNA	FC	P value
bta-miR-99b	−1.7	<0.0001
bta-miR-99a-5p	−1.0	<0.0001
bta-miR-455-5p	3.2	0.0003
bta-miR-455-3p	3.1	<0.0001
bta-miR-449c	4.1	0.00092
bta-miR-449b	16.7	0.0007
bta-miR-449a	4.0	0.00011
bta-miR-365-5p	4.5	0.00209
bta-miR-365-3p	2.7	<0.0001
bta-miR-29d	16.4	0.01292
bta-miR-29c	2.7	0.0166
bta-miR-29b	2.3	0.00317
bta-miR-29a	2.4	<0.0001
bta-miR-2483-5p	−1.6	0.0005
bta-miR-2483-3p	−2.8	0.00188
bta-miR-2285t	18.0	0.0002
bta-miR-2285k	−3.3	<0.0001
bta-miR-2285f	−1.1	0.0093
bta-miR-199c	3.2	<0.0001
bta-miR-199b	2.2	<0.0001
bta-miR-199a-5p	2.5	<0.0001
bta-miR-199a-3p	3.2	<0.0001
bta-miR-181d	2.5	0.00013
bta-miR-181c	2.6	<0.0001
bta-miR-181b	2.9	<0.0001
bta-miR-181a	3.3	<0.0001
bta-miR-146b	4.1	<0.0001
bta-miR-146a	3.2	<0.0001
bta-miR-142-5p	4.9	<0.0001
bta-miR-142-3p	16.6	0.002
bta-miR-129-5p	16.6	0.002
bta-miR-129-3p	17.8	0.002
bta-miR-10b	−1.2	<0.0001
bta-miR-107	1.9	<0.0001

*FC =  fold change in log_2_ scale. Positive FC values indicate upregulation and negative FC values describe downregulation of miRNAs at day 7 compared to day 3.*

### Temporal accumulation or degradation of miRNAs in granulosa cells of SF during the early luteal phase of the estrous cycle

The temporal miRNA expression difference in the granulosa cells of SF between day 3 and 7 of the estrous cycle was investigated to uncover the miRNAs expression dynamics occurring in subordinate follicles during the early luteal phase of the bovine estrous cycle. Results showed that only 5 miRNAs were differentially expressed between the two time points. From these, the expression level of 4 miRNAs, namely bta-miR-1271, bta-miR-100, bta-miR-424-5p and bta-miR-2285k was downregulated while the expression level of only one miRNA, bta-miR-155, was significantly upregulated in granulosa cells of SF at day 7 compared to day 3. Moreover, 4 of these 5 miRNAs, namely, bta-miR-1271, bta-miR-424-5p, bta-miR-155 and bta-miR-2285k were also differentially expressed in granulosa cells of DF between day 3 and day 7 of the estrous cycle. Therefore, 127 miRNAs were uniquely differentially expressed in DF while only one miRNA was differentially expressed in SF between day 3 and day 7 of the estrous cycle. Thus, the temporal miRNA expression dynamics in the DF was more pronounced than the SF groups between the two time points.

### Novel miRNAs detected in granulosa cells of SF and DF

In addition to known miRNAs, novel miRNAs were identified from the sequenced data. For this, we have used miRDeep2 novel miRNAs prediction tool and a sequence was considered as novel miRNA when it was not aligned to known miRNA in mirBase database. Once the sequence is confirmed as novel miRNA, it was considered as a detected when the average read counts in at least two of the biological replicates within the same sample group was ≥10 read counts. Based on this analysis, a total of 17 candidate novel miRNAs with base pair length of 17–22 were identified and detected in granulosa cells of SF or DF at day 3 or day 7 of the estrous cycle (Table 3). Of these, 10 candidates were found to be localized in the intergenic region while 7 of them were found in to be located in the intronic part of *CEP20, VCPIP1, EIFF31, ERRCC3*, *GNB1L or RPL12* genes (Table 3).

**Table 3:** Novel candidate miRNAs detected in granulosa cells of SF or/and DF at day 3 or/and day 7 of the estrous cycle.

Ids. no. miRNAs	Sequence of novel miRNAs	Ch	Precursor coordinate	DNA strand	Day 3		Day 7		Genomic location
					SF	DF	SF	DF	
*X_itw_0276*	cccccggggccgcgguuc	X	62078855..62078921	minus	11858	-	14018	-	Intergenic
*13_itw_0295*	cccggggagcccggcggu	13	38543580..38543652	minus	1190	1691	734	1096	Intergenic
*X_itw_0140*	auuggcaugucuuggaaugaag	X	30338368..30338426	minus	70	81	33	-	Intronic
*7_itw_0345*	ccgccgguccccgcccc	7	31762590..31762667	plus	57	-	-	-	Intronic (*CEP20*)
*X_itw_0247*	ugauugguacuucuuagagugga	X	29130215..29130265	plus	20	22	17	-	Intergenic
*X_itw_0078*	ugauaauacaaccugauaagu	X	82023929..82023981	minus	18	27	18	16	Intergenic
*17_itw_0191*	aaaaccggaaugaauuuuuuga	17	44700968..44701028	plus	14	12	20	-	Intergenic
*X_itw_0207*	ugauuggcauuucuuagagugga	X	29133927..29133985	plus	14	12	12	-	Intergenic
*14_itw_0201*	cggcggcggcggcgacu	14	32908534..32908603	minus	13	13	-	-	Intronic (*VCPIP1*)
*5_itw_0362*	aucccacuccugacacca	5	110183750..110183789	minus	12	44	30	13	Intronic (*EIF3l*)
*X_itw_0284*	uacugugccucgaauggguaug	X	30309767..30309823	plus	-	24	10	-	Intergenic
*24_itw_0112*	auaaccggaaugaacuuuuuga	24	6436128..6436188	plus	-	19	-	-	Intergenic
*25_itw_0146*	uccaaguaauucaggauagu	25	41470615..41470665	plus	-	17	17	-	Intergenic
*2_itw_0270*	caaaaaguucguccagauuuuu	2	5205393..5205455	plus	-	28	16	-	Intronic (*ERCC3*)
*17_itw_0056*	ggccaggggcgugucgggcucu	17	74856138..74856194	minus	-	14	-	-	Intronic (*GNB1L*)
*13_itw_0186*	agaaaaguuuguuuggguuuuu	13	73541941..73542001	minus	-	-	-	23	Intergenic
*11_itw_0182*	gccgaaagcaugggaacaggc	11	98192709..98192784	minus	-	-	-	34	Intronic (*RPL12*)

*The numbers under SF and DF columns indicate the average read counts for each novel candidate miRNAs, Ch  =  chromosome number.*

### Validation of deep sequencing data using qPCR

**Figure 11:**
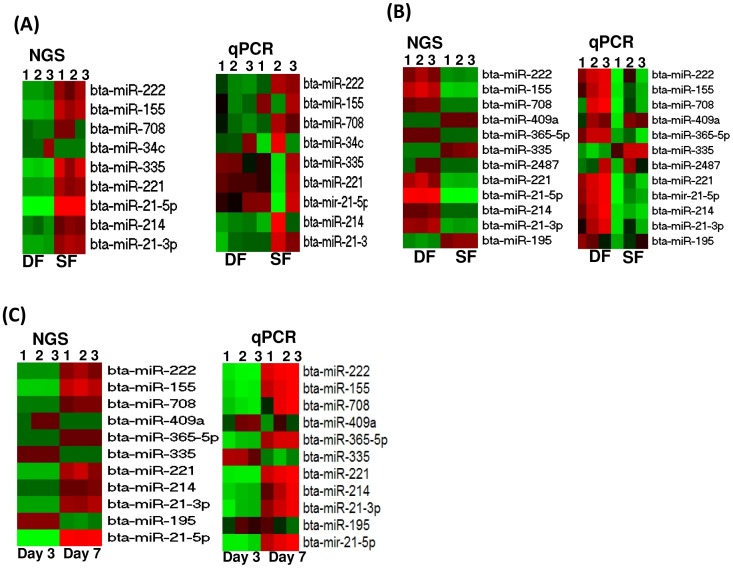
The heatmap showing the qPCR data along with the deep sequencing data for randomly selected differentially expressed miRNAs. (A) The expression pattern of candidate miRNAs in granulosa cells of SF and DF at day 3 of the estrous cycle. (B) The expression pattern of candidate miRNAs in granulosa cells of SF and DF at day 7 of the estrous cycle. (C) The expression pattern of candidate miRNAs in granulosa cells of DF at day 3 and day 7 of the estrous cycle. The red and green colours indicate high and low expression, respectively. NGS and qPCR indicate the results obtained from next generation deep sequencing and quantitative real time qPCR, respectively. Numbers, 1, 2 and 3 on the heatmaps indicate the number of biological replicates used in each sample group.

Although the results obtained by sequencing are believed to be more realistic than other methods, we opted to measure the expression level of selected candidate miRNAs using the qPCR by assuming that if the results obtained by the higher sensitive method are validated by relatively lower sensitive technique, the results obtained could be more reliable. Accordingly, the results of the qPCR data showed a similar trend of miRNA expression patterns as that of the deep sequencing result. Differences and similarities within and between biological replicates of the qPCR data along with the deep sequencing data is described in figure 11.

### Expression of differentially expressed miRNAs in theca cells

**Figure 12:**
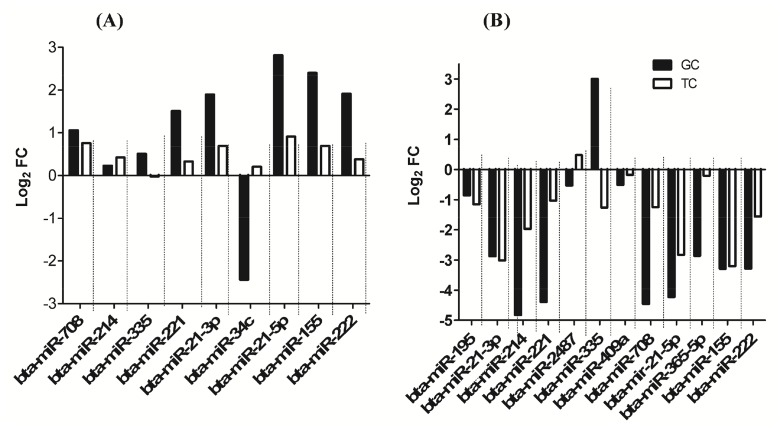
The expression patterns of candidate miRNAs in theca cells of SF and DF whose granulosa cells was used for the next generation sequencing. (A) The expression level of 9 candidate miRNAs in granulosa and theca cells of SF compared to DF at day 3 of the estrous cycle. (B) The expression level of 12 candidate miRNAs in granulosa and theca cells of SF compared to DF at day 7 of the estrous cycle. Positive and negative log_2_FC values indicate up and downregulation of miRNA, respectively in SF compared to the DF.

Following detection and identification of differentially expressed miRNAs in granulosa cells of SF and DF, the expression patterns of some miRNAs were further analysed in their corresponding theca cells both at day 3 and at day 7 of the estrous cycle. The results have shown that among 9 miRNAs analysed at day 3 of the estrous cycle, the expression of all selected miRNAs except bta-miR-34c and bta-miR-335 in theca cells exhibiting a similar trend to that of the corresponding granulosa cells (Figure 12A). Similarly, at day 7 of the estrous cycle, the expression pattern of 12 differentially expressed miRNAs in granulosa cells of SF and DF were analysed in theca cells and the results showed that the expression of selected miRNAs except miR-2487 and bta-miR-335 were exhibiting a similar expression pattern to that of the corresponding granulosa cells in SF compared to DF (Figure 12B) suggesting the companion cells of SF and DF follicles may exhibit similar expression pattern of miRNAs.

## Discussion

### MiRNAs are required to perform basal functions during folliculogenesis

One of the most significant roles of the ovary is to undergo a continuous folliculogenesis to produce viable and competent oocytes. Understanding the molecular mechanism of follicular development is essential to unravel the complex synergies orchestrated during the process of forming the fertilizable ovum. The granulosa cells are required during the major stages of oocyte growth and development including ovulation and fertilization. During folliculogenesis, the bovine follicles emerge as wave like fashion [9] by which some continue to increase in size to become dominant while others remain subordinate although all are recruited at similar time. Thus, it may be possible to imagine that the dominant and subordinate follicles have unique molecular signals that may affect differently the bidirectional crosstalk between the oocytes and the granulosa cells. Although, several authors [10]–[14] have described the presence of altered gene expression patterns in theca and/or granulosa cells of different follicular stages, the post transcriptional gene expression regulation mechanisms that lead the follicle to become dominant or subordinate are still unclear. Here we investigated accumulation or degradation of miRNAs in granulosa cells of subordinate and dominant follicles during the early luteal phase of the estrous cycle. For this, we have generated miRNA sequencing data from 12 granulosa cell samples derived from subordinated and dominant follicles both at day 3 and at day 7 of the estrous cycle using high throughput sequencing technology. Since the sequencing data were generated from three biological replicates of each sample, we believe that the analysis has been robust and the findings could be repeatable. Moreover, a specific miRNA was considered to be detected when it was expressed at least in two of the three biological replicates of each sample. Therefore, from our data, we detected several miRNAs in granulosa cells of dominant and subordinate follicles. Interestingly, 244 miRNAs were commonly detected in all samples regardless of the follicle stage and day of the estrous cycle (Figure 1B). For instance, the let-7 family members (bta-let-7f, bta-let-7a-5p, bta-let-7g and let-7i), bta-miR-10b, bta-miR-26a, bta-miR-99b and bta-miR-27b were among the highly expressed miRNAs in all follicular stages at both day 3 and day 7 of the estrous cycle. Indeed, the role of these miRNAs during folliculogenesis is not clearly known, but the let-7 family members are believed to be involved in cell proliferation by targeting multiple genes [32]. Moreover, miR-10b is reported to be involved in cell proliferation and growth by targeting the TGF-beta signaling pathway [33]. Similarly, miR-99b could function along with TGF-beta signaling pathway to influence cell migration and cell proliferation [34]. Therefore, the expression of these miRNAs both in SF and DF both at day 3 and day 7 of the estrous cycle could indicate their basal function/housekeeping cellular roles [35] during follicular recruitment, selection, dominance or follicular atresia.

### At day 3 of the estrous cycle, the granulosa cells of subordinate follicle (SF) displayed activation of miRNA compared to dominant follicles (DF)

Although, 244 miRNAs were commonly detected in all sample groups, it does not necessary imply that they were similarly expressed. Therefore, to understand significant differences in the expression of miRNAs during recruitment and selection during the early luteal phase of the bovine estrous cycle, we have performed a differential miRNA expression analysis between the granulosa cells of SF and DF at day 3 of the estrous cycle. On this day of the cycle, 14 of the 16 differentially expressed miRNAs were activated in granulosa cells of SF compared to the DF counterparts (Figure 3A). Indeed, it is unlikely to fully understand why the majority of the differentially expressed miRNAs were activated in SF compared to DF. However, similar studies in gene expression at day 3.5 of estrous cycle showed that 11 of the 16 genes measured by qPCR were found to be activated in granulosa cells of SF compared to DF [36]. In fact, the activation of miRNAs in granulosa cells of SF at day 3 of the estrous cycle could partly be linked to the repression of several genes associated with masking of the estrogen and progesterone production in granulosa cells. On this regard, it was described that at day 3 of the follicular wave, the granulosa cells of SF secreted lower estradiol levels and exhibited reduced level of FSH receptor expression compared to the granulosa cells of the DF [37]. Thus, the reduction of estradiol and follicle stimulating hormone receptor (FSH) gene expression may also be associated with downregulation of several arrays of genes potentially targeted by activated miRNAs. In line to this, the current study indicated a 2.3 to 16 fold change increase in the expression of 14 miRNAs in SF group. Among these, miR-449a and mir-449c were activated by 16 and 10.6 folds, respectively while miR-221 and miR-222 were increased by 3.5 and 6.5 folds, respectively, but only two miRNAs (miR-183, miR-34c) were found to be downregulated. Although the role of these miRNAs in folliculogenesis needs to be elucidated, the higher expression of miR-449a can be associated with growth arrests and apoptosis [38]–[40]. Similarly, overexpression of miR-221/222 is suggested to affect the growth potential of cells by inducing a G1 to S shift in the cell cycle [41]. Moreover, an increased level of miR-222 could affect the cell motility by influencing the AKT signalling pathway [42] and its involvement in human endometrial cell differentiation has been described [43]. On the other hand, miR-183 which was down regulated in the SF group is believed to have an anti-apoptotic role and it can induce cell survival [41], [44]. This may suggest while the activation of miRNAs could be associated with cell arrest, its repression might be associated with cell proliferation and survival in SF groups. Apart from this, genes targeted by those miRNAs were found to be involved in 6 main canonical pathways including Wnt signaling, TGF beta signaling, axon guidance, apoptosis and gap junction. This may imply that although further validation and verification is required, the granulosa cells in SF may exhibit downregulation of those pathways which are essential for follicular development (Figure 3B).

### The granulosa cells in subordinate follicle displayed a marked miRNA expression dysregulation at day 7 of the estrous cycle

After selection, the first dominant follicle starts to reduce the amounts of estradiol production between days 5 and 8 of the estrous cycle accompanied by emergence of the second follicular wave [45]. This phenomenon is orchestrated by up and downregulation of several arrays of genes which may have role in folliculogenesis. Thus, looking into miRNAs associated with posttranscriptional regulation of genes in subordinate and dominant follicle during this period will enhance the knowledge of molecular mechanism of folliculogenesis. In line to this, the current results revealed differences in the miRNA expression between the granulosa of SF and DF to be noticeable at day 7 than day 3 of the estrous cycle (Figures 3, 4, 7) suggesting that SF and DF could be molecularly distinguishable as the luteal phase the estrous cycle advances. This can be also explained by the fact that unlike to day 7, follicles may not reach the size of dominant at day 3 of the estrous cycle to exhibit remarkable difference between the two follicle sizes at that day of the estrous cycle. Interestingly, at day 7 of the estrous cycles, 12 miRNA families were co-activated or co-repressed in SF compared to DF groups (Table 1). For example, miR-199 family members namely, miR-199a-3p, miR-199a-5p, -miR-199b and miR-199c were repressed in granulosa cells of SF by a similar fold change (FC  = 4–7.5) indicating that these miRNAs belonging to the same family could have a similar role during folliculogenesis. Among the miR-199 family members, higher expression of miR-199a-3p was found be associated with cell proliferation by imposing G1 cell cycle arrest [46], [47]. Similarly, miR-199a-5p along with miR-30d and miR-181a is believed to induce apoptosis by targeting the *GRP78* gene [48]. On the other hand, increased expression of miR-199a significantly inhibited the ability of TGF-beta cell growth arrest and apoptosis in vitro [49] suggesting the potential involvement of miR-199 family members in cellular development.

Similar to miR-199 families, the miR-181 family members namely, miR-181a,-miR-181b, miR-181c, miR-181d were also downregulated in granulosa cells of SF. Previous study showed that miR-181a and miR-181b were found to be overexpressed in more aggressive breast cancers cells suggesting their anti-apoptotic role [50] and miR-181a can influence cellular proliferation by targeting activin receptor IIA *(ACTR2A)* [51]. In the current study, the bioinformatic analysis showed that *ACTR2A* gene was found to be potentially targeted by all these miR-181 family members. In addition, miR-181a is reported to be involved in hematopoietic lineage differentiation [52] and T-cell sensitivity and selection [53]. Reduced level of miR-181a expression was found to result in reduced glucose deprivation induced apoptosis, mitochondrial dysfunction, and loss of mitochondrial membrane potential in astrocyte cells [54] and implicated as a predictive biomarker for breast cancer metastasis and patient survival [55]. The other family member of miR-181, namely, miR-181b is also associated with cell proliferation and inhibited cell apoptosis in cervical cancer cells [56]. Thus, alteration in the expression of miR-181 family may result in dysregulation of programmed cell death and cell proliferation in granulosa cells of the subordinate follicles.

Although it is interesting to examine the functional relevance of each of the miRNAs with respect to follicular growth and development, looking into their global roles by analysing their pathway enrichment could be essential. To achieve this, we have performed a bioinformatic analysis to get insight into the functional annotation of each miRNA by predicting their potential target genes. Accordingly, we have identified several pathways including axon guidance, focal adhesion, oocyte meiosis, TGF-beta signaling, GnRH signaling, Wnt signaling, apoptosis and gap junction to be enriched by genes potentially targeted by miRNAs either enriched or repressed in granulosa cells of SF (Figures 5 and 6). Similarly, previous study on the gene expression analysis of small (≤5 mm) and large (>12 mm) follicles indicated altered expression of genes involving in TGF-beta signaling, axonal guidance and protein trafficking [57]. Moreover, the gene expression pattern of granulosa cells from small healthy (3.1±0.2 mm diameter) and atretic (4.2±0.5 mm) bovine follicles also indicated the TGF-beta signaling and apoptosis pathways to be affected in atretic follicles [7]. Indeed, the role of TGF-beta in the bi-directional crosstalk between the granulosa cells and the oocyte has been reviewed [58], [59].

In addition to the common pathways enriched by genes targeted by miRNAs increased in SF or DF, there were also unique molecular pathways enriched by genes targeted by miRNAs only enriched in the granulosa cell of DF (Figure 5). For instance, the miRNA-gene interaction and pathway analysis showed that the target genes of by miR-142-5p, namely, ENAH, ROBO1, Rho GTPases (RHOA, RHOQ), NEO1 were found to be involved in axon guidance mediated by Slit/Robo. Among these, ROBO1 is one of the four Roundabout (Robo) which is believed to be interacting with the Slits [60] and the Slit–Robo signaling can also be interacting with Rho GTPase activating proteins [61]. The guidance mediated by Slit/Robo is believed to involve in tissue growth, development, and remodelling [62]. In addition, the Slit/Robo signaling induces cell adhesion, cell proliferation and survival [63]. Given the gene expression and/or translation repression role of miRNAs, increased level of miR-142-5p in granulosa cells of DF may suggest downregulation of axon guidance by Slit/Robo pathway. Reducing the Slit/Robo activity may increase cell migration and reduce apoptosis activity [62]. Indeed, the anti-apoptosis role of miR-142-5p has been previously described [64]. This may suggest that miR-142-5p could be an important candidate for granulosa cell survival by targeting genes involving in guidance by Slit/Robo. On the other hand, the gene targeted by miRNAs enriched only in SF groups were found to be involved in O-glaycan biosynthesis, N-glycan biosynthesis, the metabolism pathways (D-glutamine and D-glutamate, vitamins and cofactors, phenyl alanine, biosynthesis of unsaturated fatty acids). The N-glycan generated by N-acetylglucosaminyltransferase is encoded by mannosyl (*alpha-1, 3*) glycoprotein beta-1, 2-N-acetylglucosaminyltransferase (*Mgat1*) [65]. Our miRNA- target-gene analysis indicated 12 genes including *MGAT1and MGAT2* to be the targets of miR-592 and miR-30e-5p, which were upregulated in granulosa cells of SF group. Previous studies, [65], [66] have shown that targeted deletion of oocyte-specific *MGAT1* gene to be associated with reduced cumulus cell number around the oocytes and reduction in ovulation rates. This may suggest aberrant expression of N-glycans could lead to abnormal folliculogenesis. In addition, lack of complex N- and O-glycans in oocytes may have a negative consequence on further embryonic development [67]. Therefore, increased level of miRNAs targeting genes involving in the N- and O-glycan biosynthesis in granulosa cells of SF groups may indicate a negative effect on follicle development in those follicular stages during the early luteal phase of the estrous cycle.

### The temporal miRNA expression dynamics is prominent in granulosa cells of dominant follicle during the luteal phase of the estrous cycle

Besides looking into the differences between SF and DF, we also attempted to understand the temporal dynamic miRNA enrichment and degradation in granulosa of SF or DF between day 3 and 7 of the estrous cycle. Results have shown that the temporal miRNA expression profile changes between day 3 and 7 of the estrous was noticeable in DF groups by which the expression profile of 131 miRNAs was found to be altered between day 3 and 7 of the estrous cycle. However, in SF groups, only 5 miRNAs were differentially regulated between these two time points. This may suggest that although both the SF and DF follicles emerged during the luteal phase and are not eligible for ovulation and both undergo regression and follicular atresia due to the influence of progesterone [68], the granulosa cells of DF exhibited marked temporal miRNA transcriptional activity alteration (Fig 9). This may suggest the presence of dynamic molecular alteration in the follicles that could be eligible for dominance. Firstly, this could be due to the fact that bovine follicles are not eligible to reach the dominant stage at day 3 unlike to day 7 when the follicle can reach dominance stage although they are not ovulated. Secondly, as the estrous cycle proceeds from day 3 to day 7, the follicles increase in size, the production of estradiol production also elevates. However, the increment of estradiol secretion usually does not occur more than 3 or 4 days and thus due to elevated of progesterone levels, the DF becomes atretic and estradiol production declines on day 6 of the estrous cycle [45]. Based on this notion, we look into the miRNAs whether their alteration is associated with estradiol production. In fact, during folliculogenesis, steroidogenesis is one of key process that happened in the granulosa cells. For this, dehydroepiandrosterone (DHEA) is produced from pregnenolone, androstenedione from progesterone by cytochrome p450 enzyme called *CYP17A1* occurs in theca cells. On the other hand, the conversion of androstenedione into estrone and conversion of testosterone into estradiol occurs in granulosa cells by aromatase gene [69]. Indeed aromatase gene (*CYP19A1*) is a validated target of miR-378 [70] and this miRNA was found to be increase in granulosa cells of DF at day 7 compared to day 3 of the estrous cycle suggesting a decrease in the level of aromatase genes expression. Similarly, the miRNAs which were found to be associated with inhibitory effect on estradiol production [71] namely, miR-96b, miR-146, miR-28, miR-29a, miR-184, miR-32, miR-34a, miR-129, miR-132, miR-133a, and miR-150 were found to be increased at day 7 of the estrous cycle. Others, miR-24, miR-122, miR-145, miR-182, miR-143 and miR-150 which increased progesterone level in human granulosa cells after they were over expressed [71] were found to be increased at day 7 of the estrous cycle. Other miRNAs which were dysregulated in DF between day 7 and day 3 of the estrous cycle namely miR-21, miR-132, miR-191 and miR-99b were also exhibited temporally expression alteration in mural granulosa cells collected from mice treated with eCG for 46 h followed by injection of hCG [72]. This may suggesting some miRNAs whose expression was repressed or enhanced in granulosa cells of DF between day 3 and 7 of the estrous may involve in steroidogenesis or they may have some role in acquisition of follicular maturation.

## Conclusion

In this study, we provided detailed insights into the miRNA enrichment and degradation in the subordinate and dominant follicle during the luteal phase of the estrous cycle using high-throughput miRNA sequencing data generated from granulosa cells obtained from subordinate and dominant follicles at day 3 and 7 of the estrous cycle. In addition to novel miRNAs, the study identified a large sets of miRNAs which are found to be differentially expressed in granulosa cells of SF and DF and suggested to play a distinct role in various physiological processes associated with the two follicle types during the course of folliculogenesis. Further detail functional analysis of clusters or families of miRNAs is needed to specifically determine the regulatory role of these noncoding small RNAs in bovine follicular development.

## Supporting Information

Table S1 **List of adapters used for Illumina library preparation.**
(DOCX)

Table S2 **RNA PCR primer sequences for TruSeq Small RNA Sample Prep Kit (Illumina).**
(DOCX)

Table S3 **The list of differentially expressed miRNAs between the granulosa cells of SF and DF at day 7 of the estrous cycle.**
(DOCX)

Table S4 **The list of significant pathways (P≤0.05) enriched by genes potentially targeted by miRNAs differentially expressed in DF between day 7 and day 3 of the estrous cycle.** (↑) indicate increased expression while (↓) depicts the reduction of miRNA expression at day 7 compared to day 3 of the estrous cycle.
(XLSX)
