# Supplementary material for: The Expression Pattern of microRNAs in Granulosa Cells of Subordinate and Dominant Follicles during the Early Luteal Phase of the Bovine Estrous Cycle
Source: PLoS One. 2014 Sep 5;9(9):e106795. doi: 10.1371/journal.pone.0106795 (PMC4156418; doi:10.1371/journal.pone.0106795)
Supplement: Table S1 — List of adapters used for Illumina library preparation. (DOCX) [file pone.0106795.s001.docx]

Table S1. List of adapter used for library preparation

| Adapters | Sequence |
| --- | --- |
| RA5 | GTTCAGAGTTCTACAGTCCGACGATC |
| RA5 -revcomp | GATCGTCGGACTGTAGAACTCTGAAC |
| RA3 | TGGAATTCTCGGGTGCCAAGG |
| RA3-revcomp | CCTTGGCACCCGAGAATTCCA |
| RTP | GCCTTGGCACCCGAGAATTCCA |
| RTP –revcomp | TGGAATTCTCGGGTGCCAAGGC |
| RP1 | AATGATACGGCGACCACCGAGATCTACACGTTCAGAGTTCTACAGTCCGA |
| RP1 -revcomp | TCGGACTGTAGAACTCTGAACGTGTAGATCTCGGTGGTCGCCGTATCATT |
|  |  |
