# Supplementary material for: The Expression Pattern of microRNAs in Granulosa Cells of Subordinate and Dominant Follicles during the Early Luteal Phase of the Bovine Estrous Cycle
Source: PLoS One. 2014 Sep 5;9(9):e106795. doi: 10.1371/journal.pone.0106795 (PMC4156418; doi:10.1371/journal.pone.0106795)
Supplement: Table S2 — RNA PCR primer sequences for TruSeq Small RNA Sample Prep Kit (Illumina). (DOCX) [file pone.0106795.s002.docx]

| Table S 2. RNA PCR primer sequences for TruSeq Small RNA Sample Prep Kit (Illumina) | | |
| --- | --- | --- |
| **Sample name** | **Index (bold)** | **Sequence** |
|  |  | \| **RNA PCR primer RP1 Sequence** \| \| --- \| \| 5’ AATGATACGGCGACCACCGAGATCTACACGTTCAGAGTTCTACAGTCCGA \| |
| A26258_S1 Index 1 (RPI1) 5’ CAAGCAGAAGACGGCATACGAGAT**CGTGAT** GACTGGAGTTCCTTGGCACCCGAGAATTCCA | | |
| A26259_S2 | Index 2 (RPI2) | 5’ CAAGCAGAAGACGGCATACGAGAT**ACATCG** GACTGGAGTTCCTTGGCACCCGAGAATTCCA |
| A26260_S3 | Index 3 (RPI3) | 5’ CAAGCAGAAGACGGCATACGAGAT**GCCTAA**GTGACTGGAGTTCCTTGGCACCCGAGAATTCCA |
| A26261_S4 | Index 4 (RPI4) | 5’ CAAGCAGAAGACGGCATACGAGAT**TGGTCA**GTGACTGGAGTTCCTTGGCACCCGAGAATTCCA |
| A26262_S5 | Index 5 (RPI5) | 5’ CAAGCAGAAGACGGCATACGAGAT**CACTGT** GTGACTGGAGTTCCTTGGCACCCGAGAATTCCA |
| A26263_S6 | Index 6 (RPI6) | 5’ CAAGCAGAAGACGGCATACGAGAT**ATTGGC**GTGACTGGAGTTCCTTGGCACCCGAGAATTCCA |
| A26267_L1 | Index 10 (RPI10) | 5’ CAAGCAGAAGACGGCATACGAGAT**AAGCTA**GTGACTGGAGTTCCTTGGCACCCGAGAATTCCA |
| A26268_L2 | Index 11 (RPI11) | 5’ CAAGCAGAAGACGGCATACGAGAT**GTAGCC**GTGACTGGAGTTCCTTGGCACCCGAGAATTCCA |
| A26269_L3 | Index 12 (RPI12) | 5’ CAAGCAGAAGACGGCATACGAGAT**TACAAG**GTGACTGGAGTTCCTTGGCACCCGAGAATTCCA |
| A26270_L4 | Index 13 (RPI13) | 5’ CAAGCAGAAGACGGCATACGAGAT**TTGACT** GTGACTGGAGTTCCTTGGCACCCGAGAATTCCA |
| A26271_L5 | Index 14 (RPI14) | 5’ CAAGCAGAAGACGGCATACGAGAT**GGAACT**GTGACTGGAGTTCCTTGGCACCCGAGAATTCCA |
| A26272_L6 | Index 15 (RPI15) | 5’ CAAGCAGAAGACGGCATACGAGAT**TGACAT** GTGACTGGAGTTCCTTGGCACCCGAGAATTCCA |
|  |  |  |
