# Supplementary material for: The Expression Pattern of microRNAs in Granulosa Cells of Subordinate and Dominant Follicles during the Early Luteal Phase of the Bovine Estrous Cycle
Source: PLoS One. 2014 Sep 5;9(9):e106795. doi: 10.1371/journal.pone.0106795 (PMC4156418; doi:10.1371/journal.pone.0106795)
Supplement: Table S3 — The list of differentially expressed miRNAs between the granulosa cells of SF and DF at day 7 of the estrous cycle. (DOCX) [file pone.0106795.s003.docx]

Table S3: Tthe list of differentially expressed miRNAs between the granulosa cells of SF and DF at day 7 of the estrous cycle

| miRNA | log2FoldChange | p value | FDR |
| --- | --- | --- | --- |
| bta-miR-2332 | 17.9 | 0.00014 | 0.00105 |
| bta-miR-409a | 16.5 | 0.00418 | 0.02198 |
| bta-miR-2446 | 16.4 | 0.01337 | 0.05797 |
| bta-miR-383 | 16.3 | 0.02067 | 0.08268 |
| bta-miR-2404 | 16.3 | 0.02350 | 0.08891 |
| bta-miR-335 | 5.5 | 0.00000 | 0.00000 |
| bta-miR-2284t-3p | 4.4 | 0.00143 | 0.00873 |
| bta-miR-6526 | 4.2 | 0.00000 | 0.00000 |
| bta-miR-190b | 3.9 | 0.00004 | 0.00035 |
| bta-miR-2344 | 3.6 | 0.00122 | 0.00753 |
| bta-miR-224 | 3.6 | 0.00000 | 0.00000 |
| bta-miR-3431 | 3.6 | 0.00000 | 0.00000 |
| bta-miR-452 | 3.3 | 0.00028 | 0.00184 |
| bta-miR-2483-3p | 3.0 | 0.00327 | 0.01769 |
| bta-miR-1343-3p | 2.8 | 0.00000 | 0.00000 |
| bta-miR-2285k | 2.6 | 0.00000 | 0.00000 |
| bta-miR-2411-3p | 2.4 | 0.00557 | 0.02774 |
| bta-miR-92b | 2.2 | 0.00000 | 0.00000 |
| bta-miR-744 | 2.1 | 0.00004 | 0.00028 |
| bta-miR-10b | 2.1 | 0.00000 | 0.00000 |
| bta-miR-2885 | 2.0 | 0.02341 | 0.08891 |
| bta-miR-505 | 2.0 | 0.00010 | 0.00079 |
| bta-miR-497 | 2.0 | 0.00000 | 0.00000 |
| bta-miR-592 | 2.0 | 0.00965 | 0.04386 |
| bta-miR-1296 | 1.9 | 0.00015 | 0.00110 |
| bta-miR-31 | 1.9 | 0.00000 | 0.00000 |
| bta-miR-574 | 1.9 | 0.00000 | 0.00000 |
| bta-miR-202 | 1.8 | 0.00000 | 0.00004 |
| bta-miR-195 | 1.7 | 0.00000 | 0.00003 |
| bta-miR-484 | 1.6 | 0.00001 | 0.00005 |
| bta-miR-339b | 1.6 | 0.00000 | 0.00000 |
| bta-miR-339a | 1.6 | 0.00000 | 0.00000 |
| bta-miR-30e-5p | 1.6 | 0.00000 | 0.00000 |
| bta-miR-1271 | 1.5 | 0.00026 | 0.00176 |
| bta-miR-6119-3p | 1.5 | 0.02644 | 0.09879 |
| bta-miR-15b | 1.5 | 0.00003 | 0.00024 |
| bta-miR-769 | 1.4 | 0.00021 | 0.00147 |
| bta-miR-30c | 1.4 | 0.00000 | 0.00000 |
| bta-miR-423-3p | 1.4 | 0.00000 | 0.00001 |
| bta-miR-92a | 1.4 | 0.00001 | 0.00006 |
| Table S3 cont.  bta-miR-128 | 1.3 | 0.00004 | 0.00034 |
| bta-miR-99a-5p | 1.3 | 0.00000 | 0.00001 |
| bta-miR-2483-5p | 1.2 | 0.02127 | 0.08447 |
| bta-miR-450b | 1.2 | 0.00623 | 0.03076 |
| bta-miR-296 | 1.2 | 0.00174 | 0.01018 |
| bta-miR-25 | 1.2 | 0.00014 | 0.00104 |
| bta-miR-16b | 1.1 | 0.00016 | 0.00113 |
| bta-miR-99b | 1.1 | 0.00221 | 0.01245 |
| bta-miR-148b | 1.0 | 0.00287 | 0.01567 |
| bta-miR-130a | 1.0 | 0.00020 | 0.00139 |
| bta-miR-2478 | 1.0 | 0.02306 | 0.08891 |
| bta-miR-143 | -1.3 | 0.00055 | 0.00353 |
| bta-miR-185 | -1.3 | 0.01498 | 0.06396 |
| bta-miR-874 | -1.4 | 0.00005 | 0.00036 |
| bta-miR-152 | -1.4 | 0.00161 | 0.00962 |
| bta-miR-22-3p | -1.4 | 0.00000 | 0.00001 |
| bta-miR-29a | -1.4 | 0.00015 | 0.00107 |
| bta-miR-486 | -1.5 | 0.00010 | 0.00077 |
| bta-miR-181d | -1.5 | 0.01942 | 0.07824 |
| bta-miR-107 | -1.5 | 0.00047 | 0.00311 |
| bta-miR-23b-3p | -1.7 | 0.00000 | 0.00004 |
| bta-miR-451 | -1.7 | 0.00002 | 0.00019 |
| bta-miR-28 | -1.7 | 0.00002 | 0.00017 |
| bta-miR-181c | -1.8 | 0.00001 | 0.00008 |
| bta-miR-3613 | -1.8 | 0.00876 | 0.04012 |
| bta-miR-2284aa | -1.8 | 0.00757 | 0.03555 |
| bta-miR-146a | -1.9 | 0.00090 | 0.00565 |
| bta-miR-24-3p | -1.9 | 0.00000 | 0.00000 |
| bta-miR-204 | -1.9 | 0.00000 | 0.00000 |
| bta-miR-365-3p | -1.9 | 0.00001 | 0.00008 |
| bta-miR-199a-5p | -2.1 | 0.00000 | 0.00000 |
| bta-miR-2425-5p | -2.1 | 0.00730 | 0.03545 |
| bta-miR-29b | -2.2 | 0.00189 | 0.01082 |
| bta-miR-181b | -2.3 | 0.00000 | 0.00000 |
| bta-miR-22-5p | -2.3 | 0.00071 | 0.00449 |
| bta-miR-199b | -2.4 | 0.00000 | 0.00000 |
| bta-miR-181a | -2.4 | 0.00000 | 0.00000 |
| bta-miR-27a-3p | -2.4 | 0.00000 | 0.00000 |
| bta-miR-326 | -2.5 | 0.00876 | 0.04012 |
| bta-miR-146b | -2.5 | 0.00000 | 0.00004 |
| bta-miR-130b | -2.5 | 0.00000 | 0.00000 |
| Table S3 cont.  bta-miR-199a-3p | -2.5 | 0.00000 | 0.00000 |
| bta-miR-149-5p | -2.6 | 0.00000 | 0.00000 |
| bta-miR-218 | -2.6 | 0.02330 | 0.08891 |
| bta-miR-214 | -2.7 | 0.00000 | 0.00000 |
| bta-miR-21-5p | -2.8 | 0.00000 | 0.00000 |
| bta-miR-199c | -2.9 | 0.00000 | 0.00000 |
| bta-miR-21-3p | -3.0 | 0.00000 | 0.00000 |
| bta-miR-23a | -3.1 | 0.00000 | 0.00000 |
| bta-miR-455-3p | -3.1 | 0.00000 | 0.00000 |
| bta-miR-222 | -3.2 | 0.00000 | 0.00000 |
| bta-miR-193b | -3.2 | 0.00000 | 0.00000 |
| bta-miR-155 | -3.3 | 0.00000 | 0.00000 |
| bta-miR-142-5p | -3.3 | 0.00354 | 0.01878 |
| bta-miR-2285t | -3.5 | 0.00857 | 0.03991 |
| bta-miR-346 | -3.5 | 0.00166 | 0.00980 |
| bta-miR-34a | -3.6 | 0.00000 | 0.00004 |
| bta-miR-150 | -3.6 | 0.00230 | 0.01281 |
| bta-miR-708 | -3.7 | 0.00000 | 0.00000 |
| bta-miR-221 | -3.7 | 0.00000 | 0.00000 |
| bta-miR-455-5p | -3.7 | 0.00021 | 0.00147 |
| bta-miR-2411-5p | -4.0 | 0.01126 | 0.05035 |
| bta-miR-1249 | -4.2 | 0.00442 | 0.02301 |
| bta-miR-483 | -4.8 | 0.02364 | 0.08891 |
| bta-miR-2389 | -18.1 | 0.02283 | 0.08891 |
| bta-miR-2487 | -18.3 | 0.01382 | 0.05946 |
| bta-miR-365-5p | -19.2 | 0.00053 | 0.00343 |
| bta-miR-184 | -23.5 | 0.00000 | 0.00000 |
